# Hypoglycemic Activity of Aqueous Extracts from *Catharanthus roseus*

**DOI:** 10.1155/2012/934258

**Published:** 2012-09-27

**Authors:** Elisa Vega-Ávila, José Luis Cano-Velasco, Francisco J. Alarcón-Aguilar, María del Carmen Fajardo Ortíz, Julio César Almanza-Pérez, Rubén Román-Ramos

**Affiliations:** División de Ciencias Biológicas y de la Salud, Universidad Autónoma Metropolitana, Avenida San Rafael Atlixco 186, Colonia Vicentina, 09340 México, DF, Mexico

## Abstract

*Introduction*. *Catharanthus roseus* (L.) is used in some countries to treat diabetes. The aim of this study was to evaluate the hypoglycemic activity of extracts from the flower, leaf, stem, and root in normal and alloxan-induced diabetic mice. *Methods*. Roots, leaves, flowers, and stems were separated to obtain organic and aqueous extracts. The blood glucose lowering activity of these extracts was determinate in healthy and alloxan-induced (75 mg/Kg) diabetic mice, after intraperitoneal administration (250 mg/Kg body weight). Blood samples were obtained and blood glucose levels were analyzed employing a glucometer. The data were statistically compared by ANOVA. The most active extract was fractioned. Phytochemical screen and chromatographic studies were also done. *Results*. The aqueous extracts from *C. roseus* reduced the blood glucose of both healthy and diabetic mice. The aqueous stem extract (250 mg/Kg) and its alkaloid-free fraction (300 mg/Kg) significantly (*P* < 0.05) reduced blood glucose in diabetic mice by 52.90 and 51.21%. Their hypoglycemic activity was comparable to tolbutamide (58.1%, *P* < 0.05). *Conclusions*. The best hypoglycemic activity was presented for the aqueous extracts and by alkaloid-free stem aqueous fraction. This fraction is formed by three polyphenols compounds.

## 1. Introduction

Diabetes mellitus is a term employed to describe a metabolic disorder characterized by persistent hyperglycemia with disturbances of carbohydrate, fat, and protein metabolism resulting from defects in insulin secretion, insulin action, or both [1]. The long-term effects of diabetes mellitus include progressive development on the specific complications of retinopathy, nephropathy, and/or neuropathy [2]. People with diabetes are at increased risk of cardiovascular disease [3].

Diabetes affects a large proportion of Mexican adults (8.18%), it is the most common cause of death in Mexico [4] and it has been estimated that close to 11.7 million Mexicans will have diabetes by the year 2025 [5].

The use of herbal medicines for the treatment of diabetes mellitus has gained importance throughout the world and there is an increased demand to use natural products with antidiabetic activity due to the side effects associated with the use of insulin and oral hypoglycemic agents [6].

*Catharanthus roseus* (L.). G. Don (Apocynaceae) is used traditionally in various regions of the world including Nigeria [6], India [6, 7], Trinidad and Tobago [8] to control diabetes. *C. roseus* (L) is an herbaceous subshrub also known as *Vinca rosea* (Linn I) [6], is native to the Caribbean Basin and has historically been used to treat a wide assortment of diseases [9]. Roots and leaves of this plant contain more than 100 alkaloids. It has economic importance from its alkaloids. The two leaf alkaloids which are most important in medicine are vinblastine and vincristine, which are used in the treatment of cancer [18]. Ajmalicine (antihypertension activity) and its oxidized form, serpentine (tranquilizer), are indol alkaloids obtained from the roots, with medicinal importance [10].

Fresh leaf juice of *C. roseus *has been reported to reduce blood glucose in normal and alloxan diabetic rabbits [7]. Moreover the antihyperglycemic and hypolipidemic effects of ethanol extract from the leaves of *C. roseus* were investigated in alloxan-induced diabetic rats [6]. Singh et al., detected the hypoglycemic activity in dichloromethane : methanol extract (1 : 1) of leaves and twigs of *C. roseus*, using streptozotocin-induced diabetic rat model [11].

In Mexico, diabetes mellitus is commonly treated with herbal extract and a total of 306 species have records of a popular use in the treatment of this endocrine syndrome. One of them is *C. roseus* that is employed as root infusion and it is commonly named as *vicaria* [12]. Its plant is also known as “teresita” or “todo tiempo”. In Mexico, it has a great distribution and it is also employed as ornamental. 

In the present study the organic and aqueous extracts from root, leaf, flower, stem, and an alkaloid-free aqueous stem fraction were evaluated for hypoglycemic activity in healthy or alloxan-induced diabetic mice. We performed a phytochemical screen of the extracts as well as the chromatographic characteristics of the alkaloid-free fraction.

## 2. Methods

### 2.1. Plant Material

The plant (rose variety) was collected in Rio Viejo, Tecuala Municipality, Nayarit, Mexico, and it was authenticated in the herbarium at the Universidad Autónoma Metropolitana. A voucher specimen is kept in this university with the reference number 70150UAMIZ.

### 2.2. Chemical Used

The alloxan and tolbutamide were obtained by Sigma Chemical Company, USA. All other reagents used in this study were analytical grade and were purchased from J. T. Baker, Mexico.

### 2.3. Extracts Preparation

The roots, stems, leaves, and flowers were obtained and they were dried and ground. The organic extracts from these parts of the *C. roseus* were obtained using a Soxhlet apparatus and solvents with different polarity: hexane, dichloromethane, and methanol (400 mL of each one). They were used in order of increased polarity. After the last organic solvent (methanol) was removed, the mark was subjected to reflux with distilled water in order to obtain the aqueous extracts.

The organic solvents were evaporated under reduced pressure meanwhile the water was removed by lyophilization. The extracts were kept to 4°C until their use. 

### 2.4. Aqueous Stem Extract Fractionation

The aqueous stem extract was fractionated to obtain a alkaloid-free fraction. The dry extract was dissolved in 0.5 M chlorhydric acid, magnetically stirring during 3 h and nonsoluble material was separated by centrifugation. The aqueous soluble portion was treated twice with chloroform, in order to obtain the acid substances and after the aqueous fraction was basified with 15% sodium hydroxide to pH 10, and free bases were also extracted with chloroform [13]. The pH of the remaining aqueous solution was adjusted with 2.0 M chlorhydric acid to pH 7.0 and the water was eliminated in a water heat bath to obtain the dry alkaloid-free fraction.

### 2.5. Animals

Male mice *Mus musculus *CD-1 strain weighing 35–45 g were used in this study. They were kept (6 per cage) according to international rules (NIH Guidelines for the handling and care of animals) as well as Mexican official standards (NOM-062-ZOO-1999). These mice were kept under automatic light and darkness cycle (12 × 12 h), as well as temperature (22 ± 1°C) and humidity controlled (humidity relative 55 ± 3°C). The animals were feed with a basic diet to rodents and water *ad libitum*. Animals were fasted for 12 h prior to the experiment, allowing access only to water and were deprived of both food and water during the experiment.

### 2.6. Diabetes Induction

After fasting 12 h, animals were rendered diabetic by injecting a freshly alloxan solution (dissolved in physiological saline, 75 mg/Kg, i.p.) after a base-line glucose estimation was done. Control mice received the same volume of saline solution. After 8 days, blood samples from the tail vein of the mice were obtained and the blood glucose levels were measured by using a glucometer (Accutrend Sensor Comfort, Roche).

### 2.7. Biological Assay

The hypoglycemic activity of the extracts and a alkaloid-free aqueous stem fraction were evaluated in healthy or diabetic mice. Animal groups were formed by 6 mice. The healthy mice were treated with the organic, aqueous extracts (250 mg/Kg, i.p.), or the vehicle (corn oil, saline solution). The diabetic mice were treated with aqueous extracts, tolbutamide, alkaloid-free aqueous stem fraction (300 mg/Kg), or saline solution. Corn oil was employed to dissolve the hexane and dichloromethane extracts meanwhile saline solution was used to dissolve the methanol extracts, aqueous extracts, tolbutamide and the alkaloid-free aqueous stem fraction.

Blood samples (approx. 0.3 mL) were collected from tail vein of the mice before and also at 2, 4, and 6 hours after treatment. Blood glucose concentrations were measured using an Accutrend Sensor glucometer (Roche).

### 2.8. Phytochemical Screening Methods

The extracts were tested for alkaloids using Mayer's, Wagner's, Bouchardt's, and Drangendorf's reagents, according to the method described by Maldoni [14]. Glycosides were determined using Benedict reagents [15] and saponins were assessed by foam test [9]. The polyphenolic compounds were tested with ferric chloride solution with chlorhydric acid and for flavonoids using 1% aluminium chloride solution in methanol, concentrated chlorhydric acid, magnesium turning, and potassium hydroxide solution [15]. They were also tested for sterols and/or terpenes using Liebermann-Burchard reaction by the methodology described previously [15]. 

### 2.9. Studies on the Alkaloid-Free Aqueous Stem Fraction

The melting point was determined (Fisher instrument). The polyphenolic and flavonoids compounds were investigated. Chromatographic analysis was performed using Waters 600 HPLC equipment with a diode-array detector (2996). The analytical separation was performed using a bondclone C18 column (300 × 4.6 mm, 10 *μ*m particle size) and eluted with a gradient of water : methanol (50 : 50) during ten minutes.

## 3. Statistical Analysis

Data are expressed as mean percent ± standard error of mean (S.E.M). Statistical comparisons were performed using one-way ANOVA, followed by Tukey Kramer test (NCSS Software). The values were considered statistically significant when *P* < 0.05.

## 4. Results

The effects of the extracts from *C. roseus* (250 mg/Kg) on blood glucose levels on healthy mice are shown in Table 1. The data are presented as mean percentage of blood glucose reduction respect to fasting concentration. The aqueous flower extract reduced the blood glucose concentration in 49.7% (2 h, *P *< 0.05), 36.64% (4 h, *P *< 0.05), and 33.13% (6 h, *P *< 0.05). The aqueous extract from leaf reduced blood glucose concentration in 50.61% (6 h, *P *< 0.05). The aqueous extract from stem lowered the blood glucose concentration in 30.16% (6 h, *P *< 0.05) and the aqueous roots extract had a blood glucose reduction of 28.10% (*P* < 0.05). The dichloromethane (CHCl_2_) flowers extract diminished the glycemic values in 28.06% (6 h, *P *< 0.05).

The organic roots extracts increased the blood glucose concentration in 40.90% (hexane, 2 h), 42.6% (CH_2_Cl_2_, 2 h), 45.4% (CH_2_Cl_2_, 4 h), and 18.11% (Methanol at 2 and 4 h). The organic stems extracts increased the blood glucose in 19.15% (CH_2_Cl_2_, 2 h) and 27.55% (methanol, 2 h). The organic leaves extracts increased the blood glucose level in 34.33% (Hexane, 2 h), 26.43% (CH_2_Cl_2_, 2 h), and 39.38% (methanol, 2 h).

Both control treatments (saline solution, corn oil) had no effects on the blood glucose concentration on healthy mice. 

The effects of aqueous extracts from *C. roseus* (250 mg/Kg), alkaloid-free aqueous stem fraction (300 mg/Kg), tolbutamide (reference drug, 125 mg/Kg), and saline solution (negative control, 6.6 mL/Kg) on alloxan-induced diabetic mice are shown in Table 2. The data are presented as mean percentage of blood glucose reduction with respect to fasting concentration.

The aqueous flowers extract decreased the blood glucose levels in 44.80% (4 h, *P *< 0.05) and 51.60% (6 h, *P *< 0.05). The aqueous roots extract lowered blood glucose concentration in 47.61% (6 h, *P *< 0.05). The aqueous leaves extract produced a maximum reduction on blood glucose of 41.95% (6 h, *P *< 0.05). The aqueous stem extract reduced in 50.72% (4 h, *P* < 0.05) and 52.94% (6 h, *P *< 0.05). The alkaloid-free aqueous stem fraction had a highest blood glucose reduction at 6 h (51.21%, *P *< 0.05). The standard drug (*tolbutamide*) had a significant blood glucose reduction (*P *< 0.05) of 40.78%, 46.92%, and 58.1% (2 h, 4 h, and 6 h resp.). Saline solution treatment had no effect on blood glucose concentrations of alloxan-induced diabetic mice.

The qualitative tests used to identify phytochemical constituents on organic and aqueous extracts from the different parts of the plant are in the Table 3. Alkaloids were detected in the dichloromethane extracts as well as the methanol extracts from flowers, leaves, stems, and roots. The aqueous root extract was also positive to alkaloid test. Terpenoids/sterols were detected in the hexane extracts from flower, leaf, and stem of *C. roseus*. They were also detected in dichloromethane extracts from leaf, stem, and root. The methanol extracts from leaf and root were positive to terpenoids/sterols test. The polyphenolic tests were positive in the methanol extract from flower, leaf, stem, and root. In the aqueous extracts from flower, leaf, and stem polyphenolic compounds were detected. Flavonoids compounds were detected in the methanol extracts from flower, leaf, stem, and root. Glycosides were detected in the methanol extracts from leaf and stem as well as in the aqueous extracts from flower and leaf. Saponins were not detected in any extracts.

The melting point of alkaloid-free aqueous stem fraction was 210–230°C. The phytochemical screening detected polyphenolic compounds. The chromatographic separation showed 3 compounds whose retention times were 3.20, 3.46, and 4.10 min, respectively. The spectral analysis for each compound is showed in Table 4. The spectral interval used was 200 at 380 nm. The first compound showed a signal at 228.3 nm. The second compound had a maximum signal at 204.6 nm and a minor signal at 302.2 nm. The third compound showed one signal at 253.2 nm.

## 5. Discussion

*Catharanthus roseus* has been used in the traditional medicine of various regions of the world to treat diabetes and the part of the plant employed is different [6–8, 12]. Due to that reason, we decided to evaluate the effect of each part of the plant (root, stem, leaf, and flower) on healthy and alloxan-induced diabetic mice.

A significant blood glucose reduction was observed in healthy mice after the intraperitoneal administration of aqueous extracts (250 mg/Kg) obtained of flower, root, stem, and leaf from *Catharanthus roseus*. The aqueous leaf extract showed the best hypoglycemic effect on healthy mice and it was time dependent. Moreover, the organic flower extracts (hexane and dichloromethane) had hypoglycemic effects on healthy mice. It is the part of the *C. roseus* that is used in Indian traditional medicine in diabetes treatment and it is taken as an infusion prepared with seven flowers/leaves [6]. The vehicles (corn oil, saline solution) used as negative control had no effect on blood glucose levels, so that the hypoglycemic effects of the extracts were due to secondary metabolites produced by *C. roseus*. 

On other hand, the aqueous extracts (250 mg/Kg) also produced hypoglycemic effects in diabetic mice. The reference drug (tolbutamide, 125 mg/Kg) reduced the blood glucose in diabetic mice, and this effect augmented gradually with time. The aqueous stem extract showed the highest hypoglycemic effect on alloxan-induced diabetic mice, so we decided to fraction it and evaluate its alkaloid-free fraction (300 mg/kg) on diabetic mice. This fraction had an important hypoglycemic effects and it was increased with time. The best hypoglycemic effect on diabetic mice was at 6 h and the ranges of blood glucose reduction were 51.21%–58.1%. 

Earlier reports indicate that dichloromethane : methanol (1 : 1) of twigs with leaves and flowers extract of *C. roseus* had significant blood glucose reduction in streptozotocin diabetic rat model [11]. The oral administration of leaf juice of *C. roseus* in healthy and alloxan-induced diabetic rabbits showed a significant antidiabetic activity and it had a more prolonged effect (at 1.0 mL/Kg) than the glibencalmide dose (40 *μ*g/Kg) in the period of 18–24 h after treatment. The authors reported that hypoglycemic effects are dose and time dependent [7] and we also observed that the hypoglycemic effects on diabetic mice are highest at a long period time. 

A previous report shows that leaves aqueous extract of *C. roseus* administrated by oral via during 15 days at single dose of 500 mg/Kg lowered the blood glucose levels on the 15th day (from 290.33–156.33 mg/dL) in streptozotocin-diabetic albino rats [16].

None of the organic and aqueous extracts from the different parts of C. roseus showed saponins presence. Earlier report indicates that alkaloids, flavonoids, terpenes, and glycosides are presented in the leaf methanol extract of C. roseus. Ohadoma and Michael are also reporting saponins in this extract [17]. Moreover, the ethanol flower extract showed alkaloids, triterpenes, tannins while saponins were not detected [9], such compounds detected as well as the saponins absence are in agreement with the results obtained by us.

These reports [9, 17] are showing discrepancy about to saponins presence. What could be the reason to this discrepancy? It could be due to the fact that C. roseus employed in these studies were grown as wild plants in different regions of the world, so this phytochemical content could be different [18]. Previous reports showed that saponins isolated from medicinal plants used in the diabetes treatment have decreased blood glucose levels of diabetic mice [19]. However, saponins are not responsible of the hypoglycemic activity in extracts from C. roseus. Ajmalicine is an alkaloid isolated from C. roseus that decreased the blood glucose levels on diabetic mice at concentration of 0.1% [19].

The alkaloid-free aqueous stem fraction (Table 4) showed compounds which have a specific spectral characteristic that absorbed light into the ultraviolet zone. The spectral characteristics as well as the phytochemical test are indicative that the alkaloid-free fraction is formed by polyphenolic compounds. Catharanthus roseus accumulate a significant number of volatile and phenolic compounds including three caffeoylquinic acids and 15 flavonol glycosides [20]. 3-O-caffeoylquinic acid as well as 4-O-caffeoylquinic acid showed three signals in their UV-Vis spectral. Such signals were at 268, 298 and 522 nm. The flavonol quercetin-3-O-(2,6-di-O-rhamnosyl) galactoside showed three signals at 255, 266 and 350 nm. The anthocyanin 1 showed three signals at 268, 298, and 522 nm meanwhile the anthocyanin 2 had three signals at 272, 300, and 538 nm [21]. A recent study showed that quercetin has hypoglycemic effects on diabetic rats [22] but this polyphenolic compound is not responsible forthe hypoglycemic activity of alkaloid-free fraction because neither of the spectral signals detected in this fraction looks like from quercetin spectral signals. On the other hand, the spectral signals of alkaloid-free fraction are different from signals reported by Ferreres et al. [21], therefore these compounds are different.

Alloxan, a beta-cytotoxin, induces chemical diabetics in wide variety of animal species through damage of insulin secreting cell [23]. Sulphonylureas like tolbutamide have been used to enhance insulin secretion in patients with type II (non-insulin-dependent) diabetes mellitus. This drug depolarizes the pancreatic beta cell and stimulates electrical activity to decrease the potassium permeability of the beta-cell membrane [24]. Alloxan-treated animals receiving the aqueous extracts (or alkaloid-free fraction) showed a best normalization of blood glucose levels in comparison to the control and this could be due to the possibility that some *β*-cells still surviving to exert their insulin realizing effect.

## 6. Conclusions

The aqueous extract (250 mg/Kg) of flowers, leaves, roots, and stems from Catharanthus roseus produced hypoglycemic effect in healthy and alloxan diabetic mice (P < 0.05). The aqueous leaf extract had the best hypoglycemic effect on healthy mice; meanwhile the best hypoglycemic effects on diabetic mice were presented by the aqueous stem extract. The alkaloid-free aqueous stem fraction had hypoglycemic effects like tolbutamide (control drug) on alloxan-induced diabetic mice. Alkaloids were detected in 10/15 extracts. None of the organic and aqueous extracts showed saponins presence. The alkaloid-free aqueous stem fraction has polyphenols compounds and its chromatographic studies showed that it is formed by three compounds.

## Figures and Tables

**Table 1:** Percentage blood glucose reduction produced by extracts from *Catharanthus roseus* after intraperitoneal administration in healthy mice.

Treatment (*n* = 6)	Dose	In fasting blood glucose (mg/dL)		Percentage blood glucose reduction	
			2 h	4 h	6 h
Control	mL/Kg				
Saline solution	6.6	55.8 ± 11.9	3.04 ± 0.37	−8.9 ± 1.051	1.43 ± 0.150
Corn oil	6.6	53.6 ± 10.2	15.61 ± 0.89	14.92 ± 0.74	22.01 ± 0.29
Flower	mg/Kg				
Hexane	250	43.2 ± 11.0	13.65 ± 1.62	27.77 ± 1.28*	15.50 ± 0.063
Dichloromethane	250	58.8 ± 15.3	−1.70 ± 0.277	22.61 ± 4.26	28.06 ± 4.06*
Methanol	250	48.4 ± 17.2	19.00 ± 3.28	22.52 ± 3.40	26.03 ± 4.00
Aqueous	250	51.3 ± 9.30	49.70 ± 3.98*	36.64 ± 3.91*	33.13 ± 2.32*
Root	mg/Kg				
Hexane	250	50.6 ± 5.10	−40.90 ± 3.36	0.592 ± 3.36	0.592 ± 0.075
Dichlorometane	250	50,0 ± 4.50	−42.6 ± 3.80	−45.4 ± 2.85	−17.6 ± 2.06
Methanol	250	50.8 ± 8.26	−18.11 ± 2.21	−18.11 ± 1.38	−17.32 ± 1.68
Aqueous	250	54.8 ± 4.91	−8.75 ± 0.41	27.33 ± 2.66*	28.10 ± 1.035*
Stem	mg/Kg				
Hexane	250	50.7 ± 10.2	−3.94 ± 1.21	11.84 ± 0.77	19.13 ± 0.75
Dichlorometane	250	52,2 ± 9.90	−19.5 ± 1.21	0.76 ± 0.059	10.53 ± 0.58
Methanol	250	50.3 ± 7.55	−27.55 ± 1.71	−5.36 ± 0.11	7.5 ± 0.539
Aqueous	250	54.7 ± 8.40	−8.95 ± 0.621	21.93 ± 1.42	30.16 ± 3.19*
Leaf	mg/Kg				
Hexane	250	53.0 ± 15.4	−34.33 ± 4.44	−6.98 ± 0.32	9.4 ± 0.53
Dichlorometane	250	52.2 ± 18.6	−26.43 ± 3.82	−11.30 ± 1.98	3.25 ± 0.24
Methanol	250	45.2 ± 10	−39.38 ± 5.61	−0.22 ± 0.090	−2.43 ± 0.266
Aqueous	250	48.6 ± 7.10	15.02 ± 2.56	26.74 ± 2.05*	50.61 ± 8.78*

Values are mean percentage blood glucose reduction (±S.E.M.). *Significant differences from glycemia in fasting (*P* < 0.05). *n* = 6.

**Table 2:** Percentage blood glucose reduction produced by aqueous extracts and a alkaloid-free fraction of *Catharanthus roseus* after intraperitoneal administration in alloxan-induced diabetic mice.

Treatment (*n* = 6)	Dose	Fast blood glucose (mg/dL)		Percent blood glucose reduction	
			2 h	4 h	6 h
Control	6.6 (mL/Kg)	542 ± 33.068	−0.21 ± 0.4	6.88 ± 1.35	−2.4 ± 0.427
Tolbutamide	125 (mg/Kg)	537 ± 36.74	40.78 ± 3.76*	46.92 ± 4.37*	58.1 ± 8.54*
Aq. flower	250 (mg/Kg)	568 ± 85	22.83 ± 1.88	44.60 ± 11.73*	51.68 ± 15.84*
Aq. root	250 (mg/Kg)	378 ± 36.74	24.29 ± 3.09	35.00 ± 2.94	47.61 ± 5.34*
Aq. leaf	250 (mg/Kg)	541 ± 40.41	5.73 ± 0.63	23.99 ± 3.90	41.25 ± 7.38*
Aq. stem	250 (mg/Kg)	391 ± 39.19	26.59 ± 6.57	50.72 ± 4.73*	52.94 ± 5.96*
Alkaloid-free stem fraction	300 (mg/Kg)	537 ± 10.20	32.57 ± 2.48	34.63 ± 2.70	51.21 ± 4.25*

Values are mean percent blood glucose reduction (±S.E.M.). *Significant differences from glycemia in fasting (*P* < 0.05). *n* = 6.

**Table 3:** Phytochemical constituents detected in *Catharanthus roseus* extracts.

Plant part	Extracts	Alkaloids	Polyphenols	Terpenoids/Sterols	Flavonoids	Glycosides	Saponins
F	Hexane	−	−	+	−	−	−
F	CH_2_Cl_2_	+	−	−	−	−	−
F	CH_3_OH	+	+	−	+	−	−
F	Aqueous	−	+	−	−	+	−
L	Hexane	−	−	+	−	−	−
L	CH_2_Cl_2_	+	−	+		−	−
L	CH_3_OH	+	+	+	+	+	−
L	Aqueous	−	+	−	−	+	−
S	Hexane	+	−	+	−	−	−
S	CH_2_Cl_2_	+	−	+	−	−	−
S	CH_3_OH	+	+	−	+	+	−
S	Aqueous	−	+	−	−	−	−
R	CH_2_Cl_2_	+	−	+	−	−	−
R	CH_3_OH	+	+	+	+	+	−
R	Aqueous	+	−	+	−	−	−

F: flower; L: leaves; S: stem; R: root; CH_2_Cl_2_: dichloromethane; CH_3_OH: methanol.

**Table 4:** Retention times and spectral signals of compounds detected in the alkaloid free aqueous stem fraction.

Compound	Retention time (min)	Wavelength (nm)	
1	3.20	228.3	
2	3.46	204.6	302.2 sh
3	4.10	253.2	

Sh: short.
